# Making the transition to workload-based staffing: using the Workload Indicators of Staffing Need method in Uganda

**DOI:** 10.1186/s12960-015-0066-7

**Published:** 2015-08-31

**Authors:** Grace Namaganda, Vincent Oketcho, Everd Maniple, Claire Viadro

**Affiliations:** Abt Associates, Lot 105 Mantsholo Road, Mbabane, Swaziland; IntraHealth International, Plot 20A Kawalya Kaggwa, Kampala, Uganda; Faculty of Health Sciences, Uganda Martyrs University, P.O. Box 5498, Kampala, Uganda; IntraHealth International, 6340 Quadrangle Drive, Suite 200, Chapel Hill, NC 27517 USA

**Keywords:** Workload, Workforce, Personnel staffing, Norms, WISN, Task sharing, Scope of practice

## Abstract

**Background:**

Uganda’s health workforce is characterized by shortages and inequitable distribution of qualified health workers. To ascertain staffing levels, Uganda uses fixed government-approved norms determined by facility type. This approach cannot distinguish between facilities of the same type that have different staffing needs. The Workload Indicators of Staffing Need (WISN) method uses workload to determine number and type of staff required in a given facility. The national WISN assessment sought to demonstrate the limitations of the existing norms and generate evidence to influence health unit staffing and staff deployment for efficient utilization of available scarce human resources.

**Methods:**

A national WISN assessment (September 2012) used purposive sampling to select 136 public health facilities in 33/112 districts. The study examined staffing requirements for five cadres (nursing assistants, nurses, midwives, clinical officers, doctors) at health centres II (*n* = 59), III (*n* = 53) and IV (*n* = 13) and hospitals (*n* = 11). Using health management information system workload data (1 July 2010–30 June 2011), the study compared current and required staff, assessed workload pressure and evaluated the adequacy of the existing staffing norms.

**Results:**

By the WISN method, all three types of health centres had fewer nurses (42–70%) and midwives (53–67%) than required and consequently exhibited high workload pressure (30–58%) for those cadres. Health centres IV and hospitals lacked doctors (39–42%) but were adequately staffed with clinical officers. All facilities displayed overstaffing of nursing assistants. For all cadres at health centres III and IV other than nursing assistants, the fixed norms or existing staffing or both fell short of the WISN staffing requirements, with, for example, only half as many nurses and midwives as required.

**Conclusions:**

The WISN results demonstrate the inadequacies of existing staffing norms, particularly for health centres III and IV. The results provide an evidence base to reshape policy, adopt workload-based norms, review scopes of practice and target human resource investments. In the near term, the government could redistribute existing health workers to improve staffing equity in line with the WISN results. Longer term revision of staffing norms and investments to effectively reflect actual workloads and ensure provision of quality services at all levels is needed.

## Background

Uganda is one of 57 countries (36 in sub-Saharan Africa) identified by the World Health Organization (WHO) as having a severe human resources for health (HRH) crisis [1]. HRH crises typically affect health worker availability, distribution and performance [2]. In Uganda, the shortage of qualified health workers, inappropriate skill mix and inequitable urban–rural distribution of health workers hinder the country’s ability to deliver basic health care services [3]. The most highly trained personnel serve relatively few; for example, with one fourth (27%) of the population, the country’s central region – which includes Kampala – employs two thirds or more of all nurses and midwives (64%), medical doctors (71%) and pharmacists (81%) [3]. Moreover, approximately 30% of all graduating doctors migrates abroad [3]. Due to limited wage provisions and the difficulty of attracting and retaining qualified health workers in rural districts, a 2010 report found that on average only 56% of approved positions were filled by appropriately trained health workers, leaving a 44% national vacancy rate [4].

To guide health worker recruitment and wage budgets and ascertain staffing levels at public health facilities, Uganda uses fixed staffing norms established in 2000 and approved by the Ministry of Public Service as part of a restructuring initiative at the local government (LG) level. The LG norms, which are determined by facility type and the scope of services expected at a given facility level, fail to account for variations in workload or output and thus are inherently inefficient. In facilities with low workloads, allocated health workers are underutilized, while in high-workload facilities, there may not be enough health workers to meet client needs. Although the norms are conservative, there is no provision to deploy health workers beyond the norms even where it is warranted by service needs and the health workers are available on the market. Moreover, the norms have remained static and are not responsive to factors that shape workload, such as population growth, geographic characteristics, changing burdens of disease and staff-intensive patient management policies. Minimum staffing levels that allow for responsive adjustments would give more power to local managers to respond to workload demands [5]. In low-technology settings such as Uganda, it is essential that local managers have the ability to recruit when need arises.

According to Uganda’s 2006 Human Resources for Health Policy, the government shall ensure that workload-based staffing norms are introduced and maintained along with the “equitable distribution of health workers over districts and health facilities on the basis of objectively established institutional needs and workloads” [6]. The Workload Indicators of Staffing Need (WISN) method, developed by the WHO in 1998, uses workload information to rationally and flexibly determine the number and type of staff required in a given health facility [7,8]. The method – which can be applied nationally, regionally or for a single health facility – is easier to use and less complex than the methods previously available and is intended to capitalize on routinely collected workload data [8]. The WHO has also developed software to facilitate WISN staffing computations. Uganda has a 10-year track record of using the WISN method at the facility and district levels in both the private not-for-profit and public sectors [9-13]. In 2011, key stakeholders recommended that the WISN method be applied at the national level.

This article describes the national WISN assessment. Its purpose was to demonstrate the key limitations of the fixed LG norms currently in use as well as generate superior evidence to inform policy on health unit staffing and staff deployment for more efficient utilization of Uganda’s available scarce human resources. Because few countries have implemented WISN nationally, there is much to be learned about how to use the results of large-scale WISN applications [14]. In the following sections, we describe Uganda’s WISN experience from a national-level perspective, presenting key findings and discussing implications that have the potential to reshape staffing policy and investments locally and in countries with a comparable HRH context.

## Methods

### Scope and setting

This national WISN assessment was conducted in September 2012 in 136 public health facilities from 33 of 112 districts. The Uganda Capacity Program (led by IntraHealth International and funded by the United States Agency for International Development) supported its implementation. To consider the application of the WISN method in Uganda, it is helpful to understand HRH responsibilities in the country’s decentralized structure (Table 1). The central Ministry of Health is responsible for development of policies, standards and guidelines as well as supervision, monitoring and evaluation. The Ministry of Health also hires staff for ministry headquarters, national vertical health programmes and regional referral hospitals. Planning, hiring and supervision for general hospitals and lower level health units (health centres II–IV) are devolved to the Ministry of Local Government at the district level, while service provision is devolved to the sub-district level (headquartered at health centres IV or general hospitals) [15]. The bulk of primary health service delivery occurs in health centres II through IV.

**Table 1:** **Ugandan health care system**

**Type of facility**	**Catchment population and size**	**Services by level of care**
Health centre II^a^	Parish (5000)	Preventive and promotive services
		Outpatient services
		Antenatal care
Health centre III	Sub-county (20 000)	All services offered at health centres II
		Maternity services
		Inpatient services (general ward)
		Laboratory services
Health centre IV	County (100 000)	All services offered at health centres III
		Inpatient services (men, women, children)
		General surgical operations
		Emergency services
		Blood transfusion
General hospital^b^	District (500 000)	All services offered at health centres IV
		Medical imaging
		In-service training
		Operational research
Regional referral hospital	Region (3 000 000)	All services offered at general hospitals
		Specialized clinics
		Specialist surgical operations
		Pathology
		Teaching
		Intern training
		Research
National referral hospital	National (10 000 000)	All services offered at regional referral hospitals
		Super-specialist clinical services
		Super-specialist surgical services
		Specialist teaching
		Research

^a^ Beneath the health centre II level are the village health teams, which carry out home visits, community mobilization and community drug distribution.

^b^ General hospitals serve a population of about 500 000 and provide a specified package of services. One hospital may serve several districts, depending on a district’s size.

### Cadres

The study used workload to determine the minimum number of each of five cadres required to deliver health services at four different levels of care (that is, health centre levels II, III and IV and general hospitals) to nationally acceptable standards. The five interdependent cadres (doctors, clinical officers, midwives, nurses and nursing assistants) have been shown to influence health cadre utilization and, hence, workload and output [16-19]. In addition, the five cadres bear the brunt of the clinical workload, have significant financial implications for the health sector due to their large numbers [20] and have been the subject of similar studies in Uganda and elsewhere, thereby facilitating local and international comparisons [9,21,22].

### Study design and sampling

The WISN method requires reliable workload information to produce accurate results [8]. For this reason, a purposive sampling strategy guided selection of the facilities included in the study. Given the study aim of demonstrating the LG norms’ limitations, the sample intentionally included only the best staffed facilities in the country (as determined by the norms). The study team expected well-staffed facilities to have more reliable data due to better capacity and to reliably reflect the typical workload for each facility type. This ensured that the results would illuminate the minimum staffing required for these facilities to function to their full capacity.

A detailed 2010 HRH audit report that compared actual staff in each facility with the LG norms showing the staffing levels of public health facilities was used to identify facilities with at least 65% of the LG norms filled with qualified staff [4]. The 65% cut-off point for a “high” staffing level was adopted because, while the national target for local government-level facilities was to move from 49% in 2010 to 75% by 2015 [15], at the time of the study – which was at the midterm of the plan – the government had assured financing for only 65% staffing levels. This purposive sampling yielded 11 hospitals, 13 level IV health centres, 53 level III health centres and 59 level II health centres (*N* = 136) spread across all 4 regions and 33 of 112 district health systems in Uganda.

### Data collection

A central WISN technical task force especially trained by the lead author (GN) spearheaded the data collection effort. It divided into five teams assigned to specific districts. In each district, the central task force teams worked with district-level teams (for example, district health officers, human resource officers, biostatisticians and health information assistants) to assemble the required data. The teams reviewed complete inpatient and outpatient data from Uganda’s health management information system (HMIS) for each sampled facility for the 1-year period from 1 July 2010 to 30 June 2011. This HMIS information was readily available because facilities report to the district level on a monthly basis. The teams also obtained current staff lists from Uganda’s human resources information system (HRIS). Informal discussions with human resource officers helped study teams interpret the staff and payroll information. After checking the monthly data for completeness and conducting quality checks, the central and district teams entered the data into Microsoft Excel to calculate annual workload and subsequently entered the workload information into the WISN software.

### WISN variables

WISN calculations require four variables: (1) activity standards, (2) available working time, (3) annual workload data and (4) current staffing. An activity standard is the time it would take a well-trained and motivated member of a particular staff category to perform an activity to acceptable professional standards. Uganda began developing national activity standards for doctors, clinical officers, nurses, midwives and nursing assistants in February 2007. The standards were set by experienced and knowledgeable professionals selected by the Ministry of Health in collaboration with district health management teams. The activity standards were field tested in March 2007, adopted as national standards in August 2007 and further reviewed and revised in 2011 [12,23-26]. The standards clearly define the roles of the various cadres. In the case of doctors and clinical officers, for example, doctors play a greater role in operating rooms, wards and maternity care. Nursing assistants in Uganda are a nonprofessional cadre trained on the job for at least 3 months in basic nursing techniques and direct patient care, who practice with or without the supervision of a qualified nurse.

The available working time – defined as the amount of time available in a year, per staff category, for delivering health services [8] – was obtained from previous WISN studies conducted in Uganda [12]. Available working time only takes into account an 8-h work day; to address the 24-h coverage provided by nurses and midwives in hospitals and health centre IV facilities, the study used previously established “individual allowance factors” to cover evening and night shifts.

The HMIS provided annual outpatient (Form 105) and inpatient (Form 108) workload data on outpatient utilization, antenatal and postnatal services, maternity services, young child clinic services, the full range of HIV services, inpatient activities, referrals and major and minor surgical procedures.

Data on current staff in the facilities were obtained from the HRIS and validated against the payroll. In assembling the data, the “doctors” category comprised all medical officers (excluding dental surgeons), “clinical officers” included all clinical officers irrespective of specialty, “midwives” included registered and enrolled midwives and “nurses” included all categories and levels (excluding midwives and nursing assistants) [4].

### Data analysis and interpretation

The study team customized the WISN software to the Ugandan context using the activity standards and available working time validated in the prior sub-national WISN studies. Using annual workload data and data on current staffing, the WISN software generates several indicators that are vital for decision-making. The WISN difference (current staff − required staff) shows the magnitude of staffing gaps or overstaffing: a negative value signifies a shortage, and a positive value represents an excess in staffing. The WISN ratio (current/required staff), on the other hand, is an indicator of workload pressure and is key to decisions about prioritizing staffing. Using the WISN ratio, workload pressure calculations were derived using the following formula: [1 − WISN ratio] * 100. We interpreted workload pressure according to the classification developed by investigators in Indonesia, who defined pressure as ranging from “low” (1-29%) to “high” (30-40%), “very high” (41-60%) or “extremely high” (>60%) [27].

## Results

Table 2 expresses existing staffing levels as a percentage of the staffing requirements ascertained by the WISN method. According to this comparison, all three types of health centres had shortages of nurses and midwives, with only 42% to 70% of required nurses and 53% to 67% of needed midwives. The two higher level facilities (health centre IV and general hospital) had significantly fewer doctors than needed (39-42%) but more than adequate staffing of clinical officers. All facility types displayed overstaffing of nursing assistants.

**Table 2:** **Current staffing as a percentage of WISN requirements, by type of health facility**

**Type of health facility (** ***N *** **= 136)**	**Doctors (%)**	**Clinical officers (%)**	**Midwives (%)**	**Nurses (%)**	**Nursing assistants (%)**	**Average (%)**
Health centre II	–	–	67	70	167	101
Health centre III	–	56	62	42	145	76
Health centre IV	39	140	53	52	191	95
General hospital	42	113	126	134	119	107

**Figure 1:**
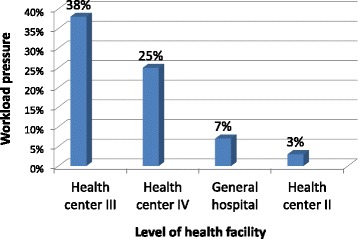
Workload pressure for all cadres combined, by level of health facility.

**Figure 2:**
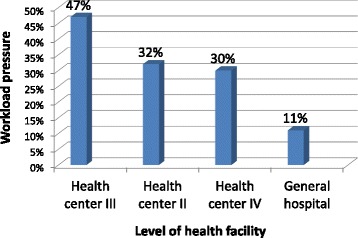
Workload pressure for qualified health workers, *Excluding nursing assistants by level of health facility.

Workload pressure varied according to type of health facility. For all staff categories combined (Figure 1), workload pressure was high (38%) at health centre III facilities. Workload pressure at health centre IV facilities (25%), although in the low category, was four to eight times greater than at health centre II facilities (3%) and hospitals (7%). When the workload pressure calculations were adjusted to exclude nursing assistants and include only qualified health workers (Figure 2), the workload pressure was very high in health centre III facilities (47%) and high in health centres II (32%) and IV (30%), while remaining low in hospitals (11%).

Table 3 shows workload pressure by individual cadre for each facility type. Workload pressure was very or extremely high (58-61%) for doctors and ranged from high to extremely high (30-58%) for nurses at nearly all facility types. Similarly, workload pressure was high or very high for midwives (33-47%). Workload pressure for clinical officers varied, with no pressure at the health centre IV and hospital levels but very high pressure (44%) at health centre III facilities. A negative workload pressure, as shown for nursing assistants at all facility levels, indicates that more staff members are available in that cadre than required for the workload experienced.

**Table 3:** **Workload pressure, by cadre and facility level**

**Type of health facility (** ***N*** ** = 136)**	**Doctors (%)**	**Clinical officers (%)**	**Midwives (%)**	**Nurses (%)**	**Nursing assistants (%)**	**All cadres (%)**
Health centre II	–	–	*33* ^a^	*30* ^a^	−67	3
Health centre III	–	*44* ^b^	*38* ^a^	*58* ^b^	−45	*38*
Health centre IV	*61* ^c^	−40	*47* ^b^	*52* ^b^	−91	25
General hospital	*58* ^b^	−13	−26	23	−43	7

^a^High workload pressure.

^b^Very high workload pressure.

^c^Extremely high workload pressure.

Table 4 compares existing staffing, the LG norms and WISN requirements for each cadre and facility level for the 136 facilities combined. A negative difference between the current staffing and the WISN requirements (“current − WISN” column) reflects a staffing shortage for the workload experienced at that level of the health facility. Table 4 indicates that whereas nursing assistants are currently available in excess of need, the other cadres are almost universally in shortage at the three levels of health centres. In comparing the LG norms with the WISN requirements (“norm − WISN” column), a negative result means that the norms recommend fewer numbers of staff than required. Table 4 shows that this again is true for all qualified health workers (excluding nursing assistants) at the three types of health centres. We discuss the Table 4 findings by facility type in the following paragraphs.

**Table 4:** **Staffing, LG norms and WISN requirements, by cadre and type of health facility**

**Facility/cadre**	**Current**	**LG norms**	**WISN**	**Current − WISN**	**Norm − WISN**
Health centre II (*n* = 59)					
Nursing assistants	92	118	55	37	63
Nurses	48	59	69	*−21*	*−10*
Midwives	45	59	67	*−22*	*−8*
Health centre III (*n* = 53)					
Nursing assistants	157	159	108	49	51
Nurses	168	212	397	*−229*	*−185*
Midwives	104	106	169	*−65*	*−63*
Clinical officers	85	53	151	*−66*	*−98*
Health centre IV (*n* = 13)					
Nursing assistants	111	65	58	53	7
Nurses	100	104	207	*−107*	*−103*
Midwives	66	52	124	*−58*	*−72*
Clinical officers	56	26	40	16	*−14*
Doctors	14	26	36	*−22*	*−10*
General hospital (*n* = 11)					
Nursing assistants	406	165	284	122	*−119*
Nurses	476	803	356	120	327
Midwives	230	308	183	47	125
Clinical officers	99	88	88	11	0
Doctors	39	121	92	**−53**	29

### Health centre II facilities

As Table 4 reveals, the LG norms had not been achieved for any of the cadres across the 59 facilities sampled in this category, and existing staffing was also lower than the WISN requirements (with the exception of nursing assistants). The LG norms actually overprescribe nursing assistants, establishing more than twice as many positions as the workload-derived WISN requirement indicates are needed.

### Health centre III facilities

In the 53 health centre III facilities, Table 4 shows that norm provisions had essentially been met for all cadres except nurses. However, both the norms and existing staffing were below the WISN requirements for nurses, midwives and clinical officers. To respond to the general inadequacy of the norms, some health centre III facilities had actually pursued a formal process to trade off other positions (presumably nurses) in order to recruit clinical officers above the norms’ provisions, with 85 clinical officers in post versus 53 approved positions (one per facility).

### Health centre IV facilities

The 13 health centre IV facilities had also generally achieved or exceeded the LG norms (with the exception of doctors), but the LG norms were again inadequate in comparison with the WISN requirement (Table 4). Health centre IV facilities require twice as many nurses and midwives as the number prescribed by the norms and about three doctors per facility versus the two recommended by the norms. The need for doctors is particularly high in districts without general hospitals, because the workload in those districts is higher.

### Hospitals

The 11 general hospitals had a substantial shortage of doctors according to the LG norms. Although the LG norms allow for more staff than hospitals require according to the WISN estimates, it is possible that the hospitals’ workload was artificially low due to the shortage of doctors. This is because the presence or absence of doctors influences the workload of the other cadres (for example, nurses and midwives) that work alongside doctors.

Finally, the study derived minimum standards to respond to policymakers’ preference for fixed norms for planning and budgeting purposes. WISN averages were calculated to determine the most appropriate minimum staffing for each level of health facility to provide the expected range of services. Table 5 compares the WISN averages with the LG norms and indicates whether the norms understate or exceed the WISN requirements. Means were used because, overall, the WISN results did not vary much for facilities of the same type. (The one exception was health centre IV facilities in districts lacking hospitals, where the health centres often function as hospitals, with higher workloads but without a corresponding and necessary adjustment to staffing norms.)

**Table 5:** **LG norms and WISN average requirements per facility, by cadre and type of health facility**

**Facility/cadre**	**LG norms**	**WISN average**	**Norms versus WISN average**
Health centre II			
Nursing assistants	2	1	Norm > WISN
Nurses	1	1	Norm = WISN
Midwives	1	1	Norm = WISN
Health centre III			
Nursing assistants	3	2	Norm > WISN
Nurses	4	7	*Norm < WISN*
Midwives	2	3	*Norm < WISN*
Clinical officers	3	3	Norm = WISN
Health centre IV			
Nursing assistants	5	5	Norm = WISN
Nurses	8	12	*Norm < WISN*
Midwives	4	10	*Norm < WISN*
Clinical officers	2	3	*Norm < WISN*
Doctors	2	3	*Norm < WISN*
General hospital			
Nursing assistants	15	16	*Norm < WISN*
Nurses	73	36	Norm > WISN
Midwives	28	17	Norm > WISN
Clinical officers	8	8	Norm = WISN
Doctors	12	9	Norm > WISN

Examining Table 5, the LG norms for the nurse and midwife cadres at *health centre II* facilities are in basic agreement with the WISN averages. At the *health centre III* level, however, the LG norms are less than the WISN average for nurses and midwives. This means that even with 100% achievement of the LG norms, these cadres would be working under pressure. At the *health centre IV* level, the LG norms for clinical officers and doctors are below but closer to the WISN averages. However, the norms for nurses and midwives again are grossly inadequate – approximately half of what is required based on the typical workload at this type of facility. For example, health centre IV facilities handle an average of 6 deliveries and 24 antenatal care (ANC) appointments per day. The four midwives recommended by the LG norms are insufficient for this workload, particularly given that additional activities beyond ANC and maternity duties include outreach, home visits, meetings, management activities and continuing medical education. The WISN requirement of at least 10 midwives for a health centre IV would ensure 24-h maternity coverage, acceptable-quality service provision and personal leave. Similarly, the LG norms’ provision of 8 nurses for health centre IV facilities is inadequate to meet the average daily workload of 90 outpatients, 10 inpatients, 6 admissions, 20 HIV counselling and testing appointments and 5 operations. According to the WISN calculations, health centre IV facilities should have at least 16 nurses to be able to provide 24-h coverage, carry out additional support and administrative work and take leave when needed. Finally, at the *general hospital* level, the LG norms agree with the WISN requirement for clinical officers but are high compared with the WISN requirements for doctors, midwives and nurses.

## Discussion

The HRH shortfall in Uganda highlights the importance of identifying innovative ways to maximize efficient use of scarce human resources in the health sector, particularly in the context of population growth, increased demand for services and changing disease management policies. Through the comparison of national WISN results with existing staffing norms, the WISN method offers a useful policy tool, demonstrating the inadequacies of existing staffing norms in public sector facilities. This study built on a number of smaller scale WISN efforts that began in 2004 [9-13]. Use of WISN results has already been successful in the private not-for-profit sector due to strong leadership and support as well as interest in continuous quality improvement [28]. The lengthy track record of WISN use, the rigorous steps taken to validate national activity standards and the ongoing data quality checks produced accurate and trustworthy findings that provide a sound evidence base to reshape policy and adopt more flexible workload-based norms that can be used to plan recruitment and wage budgets at the district level.

In light of the WHO’s recommendation that WISN assessments be repeated every 3 to 5 years [8], the need to review the outdated LG norms while taking the WISN-derived workload findings into account cannot be overemphasized. This is particularly critical for health centres III and IV, which have the most acute human resource shortages but at the same time are having more services (especially HIV care and maternal and child health services) moved to their level [29-31]. HIV-related services encompass HIV testing and counselling, prevention of mother-to-child transmission, safe male circumcision and provision of antiretroviral therapy. Where districts lack general hospitals and health centre IV facilities are functioning by default as hospitals, with correspondingly higher workloads, the need for workload-based staffing norms is even more pressing and requires that those health centres essentially be staffed as hospitals. This is only possible if a flexible approach to staffing is permitted.

The study’s inclusion of only facilities staffed at 65% or more of the LG norms vividly demonstrates that even in facilities that are considered to be well staffed, their staffing levels are not adequate to provide the full range of services expected and to handle the accompanying workload. Therefore, in facilities and districts with staffing below 65%, the HRH situation is even more challenging. This clearly demonstrates the need to review the use of LG norms if the health sector is to equitably provide at least the minimum health care package.

Where health workforce shortages and skill mix imbalances prevail, task shifting has sometimes been put forth as a policy option [32]. Earlier applications of the WISN method in Uganda observed that task shifting was occurring unofficially in response to high workload pressure, with nursing assistants stepping in for nurses and midwives and clinical officers filling in for doctors [9,11,28]. However, in the absence of any official review of professional scopes of practice, and without adequate training and supervision, task shifting is inappropriate and raises concerns about quality of care [9,32] and “task piling” or “dumping of tasks to others” [33]. The evidence generated by the WISN application in Uganda could be useful in supporting a more integrated model of care that enables task sharing, fosters teamwork and promotes an appropriate and diversified skill mix [34].

In Uganda, new schemes of service for nurses have recommended a gradual phaseout of the nursing assistant cadre. However, as the study results suggest, nursing assistants play a significant (even if not well-supervised) role in service delivery and provide support services to professional health workers at an affordable cost. As our analyses of workload pressure showed, eliminating the nursing assistant cadre would have considerable implications for the workload of already-stretched nurses and midwives. The WISN method could be used to estimate the increased number of nurses that would be required to assume nursing assistants’ duties, consider the implications in terms of HRH investments in training and recruitment and help the Ministry of Health determine whether phasing out nursing assistants would provide a cost-effective solution conducive to quality care.

In Uganda’s decentralized health care system, districts are devolved from the centre, meaning that decisions about health worker deployment take place at the district level. Because some districts have persistently failed to attract doctors, there is arguably a need for the Ministry of Health to retain central authority over the deployment of certain cadres such as medical officers and specialists. This would enable interdistrict transfers and improve service coverage nationally. Although such a step might meet with resistance on the grounds that it reverses the policy of decentralization, the national health goal of equity in access to health care is a stronger value and a necessary step toward attainment of universal health coverage. The Health Sector Strategic and Investment Plan highlights the need for greater equity in its recommendation to recentralize recruitment and deployment of critical cadres [15]. Moreover, centre-led deployment of staff should be quite feasible given that the payment of salaries is already centralized. There is a need for dialogue among stakeholders to consider how to balance the goals of decentralization and equitable distribution of health workers.

These WISN results highlight several opportunities to redistribute staff. For example, in districts with general hospitals, it might make sense to redistribute staff from the hospitals to understaffed health centres. The workload-based findings could also guide transfers of staff from underutilized health centres to those with high workloads, with the aim of improving service coverage and increasing efficient use of scarce skills. However, these opportunities are hampered by audit mechanisms that use the LG norms as the reference for staffing. As the health sector begins to advocate for the adoption of workload-based norms, it will be important for the Ministry of Health to work with entities such as the Auditor General’s office and the Health Services Delivery and Monitoring Unit to agree on a way forward.

Uganda’s experience furnishes some lessons about how to successfully apply the WISN method (summarized in Table 6). Most importantly, perhaps, systematic planning is necessary to foster a culture of workload-based human resource management. Establishing well-defined roles and responsibilities for WISN applications – with clear deliverables, timelines and reporting structures – is vital in this regard. In Namibia, for example, a task team reported to the Permanent Secretary and provided monthly updates to a national restructuring committee [14]. In Uganda, the national WISN application was slowed by turnover of technical and senior managers as well as limited ownership and stewardship of the process. The average tenure of senior leaders in African government ministries is just 3.9 years, and over half of African ministers of health turn over as often as every 2 years [35]. Comparable turnover among senior managers can affect WISN implementation because of the loss of institutional memory and the disruption to functioning teams [36]. While some high-level Ministry of Health leadership is needed to ensure political oversight and follow-through on important decisions, the ideal WISN steering committee should include district-level health managers and professionals who report to senior ministry management. Uganda is fortunate to have been able to institutionalize WISN training within the country’s master’s-level health service management degree programme, which ensures a steady supply of managers knowledgeable about the WISN method and working at different levels of the health system.

**Table 6:** **Lessons learned about applying the WISN methodology in Uganda**

**Focus**	**Lessons**
Stakeholder involvement	• Involve key stakeholders early in process
	• Include stakeholders who will use WISN results (for example, district and hospital managers, policymakers)
Leadership and technical team	• Ensure presence of national steering committee appropriately housed and led by a high-level policymaker
	• Establish multiskilled technical team comprising human resource managers, health professionals, health information officers, information technology experts and WISN experts
Data sources and reporting systems	• Understand reporting system
	• Ascertain availability, definitions and location of workload and human resource data
	• Target correct data sources and use data appropriately in WISN calculations
	• Improve timeliness and quality of human resources information system reporting, including training data managers
	• Establish interoperability of WISN software with existing workload reporting systems
WISN expertise	• Ensure training on correct use of the WISN tool
	• Allow adequate time for WISN application
	• Ensure clear understanding of each staff category to define appropriate activity standards

### Limitations

The WISN methodology relies heavily on workload data and hence is influenced by the availability, quality and precision of workload data [8,13]. This can be an important issue in the credibility of the recommendations. In this study, the data were validated by multiple checks. General hospitals appeared to have more staff than recommended by the WISN requirements (107%). However, the relatively low WISN requirements for hospital nurses and midwives may be due to the understaffing of doctors in all the hospitals studied. This is because workload for nursing cadres tends to be generated by doctors (for example, through the wider scope of services offered by doctors, more tests ordered or more support required for surgical procedures). In hospitals where there are no or few doctors, the workload for nurses and midwives is likely to be affected, thereby lowering their workload-based estimates. Given the shortage of doctors in this study (42%), hospital nurses and midwives were likely underutilized despite the appearance of overstaffing. Thus, the findings pertaining to general hospitals may need to be interpreted with caution.

The WISN method is well suited to determine the minimum staffing mix needed to deliver expected services at a given facility. However, our experience in Uganda also suggests the need to set staffing requirements that fully reflect the service package expected at each level of health facility and the appropriate technical teams to deliver the entire package of services, even if workload is low. By taking into account the service package expected at a given facility level, a particular cadre might be recommended irrespective of the WISN results.

## Conclusions

Uganda’s current staffing norms do not represent the optimal value for HRH investments because they are not responsive to actual needs and do not encourage deployment of health workers where they are needed most. One goal should be to continue to build capacity for in-depth health workforce analysis – including reliable workforce and service data management systems – to generate the kind of evidence needed to guide domestic and development partner investments in health worker production, hiring and deployment and align these to national and local needs. This type of analysis could inform the adoption of policies that, for example, establish rural recruitment quotas, expand the intake of rural-origin training candidates, institute incentives for working in underserved areas or promote good working conditions that enhance retention [37].

Domestically, the Ugandan government previously had committed to increasing funding for health centre staffing to 75% of the LG norms over a 5-year period. In 2012, however, extensive advocacy efforts resulted in a high-level policy shift that emphasized funding staff of health centres III and IV at 100%. Although “the crisis in gross understaffing and absenteeism facing the public health sector” remains a major challenge [38], the policy climate is nonetheless auspicious for drawing attention to the WISN results and progressively raising recruitment targets to the level of the WISN requirements.

In this context, the WISN findings have several broad implications that can help guide HRH investments by government as well as health and development partners. First, in the short term, the government of Uganda should foster a policy environment that enables redistribution of existing health workers toward greater equity in line with the WISN results. This will require strong government leadership and oversight to maintain reliable service statistics and actively and flexibly manage staff deployment as workloads change. Both government and development partners must carefully balance their HRH investments, however, enhancing capacity in the busiest health facilities to cope with growing workloads while also taking steps to develop capacity at less busy health facilities to improve utilization of services by the catchment population. To be avoided is an imbalanced scenario where health personnel are concentrated in busier health units serving a particular geographic area but are reduced in less frequented health units, compromising quality of care in the latter and triggering further declines in utilization of services.

The results highlighting discrepancies between Uganda’s actual staffing, the LG staffing norms and the workload-based WISN requirements also suggest that it is probably futile to apply the WISN method below a certain staffing threshold. When staffing levels are far below the minimum required to provide services of reasonable quality, the government and development partners should instead focus on increasing investments in health worker recruitment to reach current staffing standards, even if the latter are inadequate when examined with a WISN lens. In resource-constrained settings where governments are unable to immediately mobilize sufficient funds to hire additional health workers or are hampered by cumbersome hiring procedures, development partners can support short-term secondment or rapid hiring strategies to quickly attract health workers to government health facilities as has been done in Botswana and Kenya [39-42]. This type of approach can be successful in addressing workforce shortages but requires significant investment on the part of the partner institution as well as a firm government commitment through appropriate agreements to absorb the seconded or contracted health workers after the period of external support comes to a close [39]. In Kenya, emergency and rapid hiring programmes that rapidly deployed qualified health workers to understaffed public health facilities have been able to successfully transition many of the contracted health workers to county government payrolls [40-42].

For the longer term, the government of Uganda should review and revise the staffing norms to effectively reflect actual workloads and ensure provision of good quality services at the various levels of care. It bears repeating that while the LG norms were generally lower than the WISN requirements, actual staffing levels for the majority of the cadres studied were even lower. One of the reasons for the understaffing is that the current level of HRH investment is inadequate to fill all open positions. As the government reviews the LG staffing norms and gradually aligns them with the WISN results, both the government and development partners will be called on to develop sustainable medium- and long-term investment strategies to significantly increase staffing beyond present levels. To this end, the WISN results have the potential to be useful as an objective tool to facilitate accurate staffing levels, maximize efficient use of scarce resources and promote improved service coverage across district health systems.

## Abbreviations

ANC: Antenatal care
HMIS: Health management information system
HRH: Human resources for health
HRIS: Human resources information system
LG: Local government
WHO: World Health Organization
WISN: Workload Indicators of Staffing Need
